# Supplementary material for: Structure, function and assembly of soybean primary cell wall cellulose synthases
Source: eLife. 2025 May 14;13:RP96704. doi: 10.7554/eLife.96704 (PMC12077881; doi:10.7554/eLife.96704)
Supplement: Supplementary file 1. [file elife-96704-supp1.docx]

**Supplemental File 1.** Cryo-EM data collection, refinement, and validation statistic.

|  | GmCesA1  (EMDB-43244)  (PDB: 8VHZ) | GmCesA3  (EMDB-43241)  (PDB: 8VHT) | GmCesA6  (EMDB-43245)  (PDB 8VI0) |
| --- | --- | --- | --- |
| **Data collection and processing** |  |  |  |
| Microscope | Titan Krios | Titan Krios | Titan Krios |
| Camera | GIF K3 | GIF K3 | GIF K3 |
| Magnification | 81,000 | 81,000 | 81,000 |
| Voltage (kV) | 300 | 300 | 300 |
| Electron exposure (e–/Å^2^) | 50 | 50 | 50 |
| Defocus range (μm) | -1.1 to -2.2 | -1.1 to -2.2 | -1.1 to -2.2 |
| Pixel size (Å) | 1.08 | 1.08 | 1.08 |
| Symmetry imposed | C3 | C3 | C3 |
| Initial particle images (no.) |  |  |  |
| Final particle images (no.) | 181,101 | 237,568 | 192,666 |
| Map resolution (Å)  FSC threshold | 3.3  0.143 | 3.2  0.143 | 3.0  0.143 |
| Map resolution range (Å) | 3.3 - | 3.2 - | 3.0 - |
|  |  |  | ­­ |
| **Refinement** |  |  |  |
| Initial model used | AlphaFold generated | AlphaFold generated | AlphaFold generated |
| Map sharpening *B* factor (Å^2^) | -117.5 | -105.1 | -92.4 |
| Model composition  Non-hydrogen atoms  Protein residues  Ligand | 17190  2139 | 16644  2064  BGC | 17694  2172  BGC |
| Mean *B* factors (Å^2^)  Protein  Ligand | 39.47 | 56.40  85.27 | 38.03  72.40 |
| R.m.s. deviations  Bond lengths (Å)  Bond angles (°) | 0.003  0.603 | 0.002  0.53 | 0.002  0.499 |
| Ramachandran plot  Favored (%)  Allowed (%)  Disallowed (%) | 96.88  3.12  0.00 | 97.05  2.95  0.00 | 97.7  2.3  0.00 |
| Validation  MolProbity score  Clashscore  Poor rotamers (%) | 1.52  6.32  0.16 | 1.42  4.91  0.00 | 1.58  6.87  0.00 |
| Overall correlation coefficients  CC (mask)  CC (peaks)  CC (volume) | 0.76  0.62  0.74 | 0.82  0.79  0.66 | 0.85  0.74  0.83 |
